# Identifying Protein Complexes With Clear Module Structure Using Pairwise Constraints in Protein Interaction Networks

**DOI:** 10.3389/fgene.2021.664786

**Published:** 2021-08-27

**Authors:** Guangming Liu, Bo Liu, Aimin Li, Xiaofan Wang, Jian Yu, Xuezhong Zhou

**Affiliations:** ^1^School of Computer Science & Engineering, Xi'an University of Technology, Xi'an, China; ^2^Hebei Key Laboratory of Agricultural Big Data, College of Information Science and Technology, Hebei Agricultural University, Baoding, China; ^3^Beijing Key Lab of Traffic Data Analysis and Mining, School of Computer and Information Technology, Beijing Jiaotong University, Beijing, China

**Keywords:** PPI, protein complex, NMTF, module structure, must-link constraint

## Abstract

The protein-protein interaction (PPI) networks can be regarded as powerful platforms to elucidate the principle and mechanism of cellular organization. Uncovering protein complexes from PPI networks will lead to a better understanding of the science of biological function in cellular systems. In recent decades, numerous computational algorithms have been developed to identify protein complexes. However, the majority of them primarily concern the topological structure of PPI networks and lack of the consideration for the native organized structure among protein complexes. The PPI networks generated by high-throughput technology include a fraction of false protein interactions which make it difficult to identify protein complexes efficiently. To tackle these challenges, we propose a novel semi-supervised protein complex detection model based on non-negative matrix tri-factorization, which not only considers topological structure of a PPI network but also makes full use of available high quality known protein pairs with must-link constraints. We propose non-overlapping (NSSNMTF) and overlapping (OSSNMTF) protein complex detection algorithms to identify the significant protein complexes with clear module structures from PPI networks. In addition, the proposed two protein complex detection algorithms outperform a diverse range of state-of-the-art protein complex identification algorithms on both synthetic networks and human related PPI networks.

## 1. Introduction

Specific biological functions are usually carried out by a group of interacted proteins rather than by a single protein in human cells. In the past few decades, a large amount of protein-protein interactions (PPI) have been exploited with the development of a broad range of high-throughput experimental technologies, such as two-hybrid systems (Ito et al., 2001) and mass spectrometry (Aebersold and Mann, 2003). However, there is plenty of scientific knowledge that remains to be uncovered from PPI networks beyond sole interactions between proteins. A tacit assumption is that protein pairs with similar links are considered to perform similar functions which paves the way of exploring protein complexes. Generally, PPI networks have diverse topological properties such as module structure (Wagner et al., 2007), which possess numerous densely connected. Then, a large number of researchers focus on splitting a PPI network into dense groups such as protein complexes (Zahiri et al., 2020). Therefore, identifying protein complexes accurately is fundamental to demonstrating the underlying biological processes within cells. In recent years, large amounts of computational algorithms have been presented to detect protein complexes automatically from PPI networks in computational biology fields (Ashtiani et al., 2018).

As we all know, a PPI network is usually formulated as an undirected graph with weighted or un-weighted edges in accordance with the protein-protein interaction data. In general, proteins are treated as nodes and interactions act as edges in a graph. Since proteins that interact with each other are thought to be more likely to execute similar biological function to those non-wired proteins within PPI networks, thus, there are more than one tightly linked regions in a graph which are empirically considered as protein complexes (Spirin and Mirny, 2003; Tadaka and Kinoshita, 2016). The problem of detecting protein complexes can be regarded as a community detection issue in the complex network field. Therefore, it is a popular way to make use of classical clustering algorithms or community detection methods to discover potential modular structures within which proteins that are densely linked to each other are treated as protein complexes from PPI networks (Bader and Hogue, 2003; Liu et al., 2019).

In recent decades, numerous computational algorithms have been exploited to identify protein complexes from PPI networks. These methods can generally be grouped into two categories: unsupervised and supervised (or semi-supervised). Many unsupervised methods have been proposed to detect non-overlapping protein complexes from PPI networks, such as the betweeness-based algorithm proposed by Holme et al. (2003), shortest path based approach devised by Arnau et al. (2005) and other hierarchy-based clustering algorithms (He and Chan, 2018). Furthermore, proteins often participate in more than one complex to accomplish various functions; therefore, methods have been developed to consider overlaps among protein complexes (e.g., MCODE Bader and Hogue, 2003, CFinder Adamcsek et al., 2006, and ClusterONE Nepusz et al., 2012). However, these unsupervised algorithms solely focus on the topological structures of PPI networks. Currently available PPI networks are sparse because only a small subset of interactions between protein pairs generally occur (e.g., fewer than 20% of the possible links have been exploited in human cells) (Menche et al., 2015). Furthermore, considerable amounts of false positive interactions exist in sparse PPI networks, such that the proportion of false positive edges between proteins may approach 50% (Von Mering et al., 2002). These limitations have been the dominant obstacles to the discovery of specific protein complexes from PPI networks (Xu et al., 2018). Thus, the protein complexes detected by the existing unsupervised protein complex predicted algorithms, which solely focus on PPI network topological structure, may have limited accuracy (Yao et al., 2019).

Fortunately, substantial high-quality hand-curated protein complex data are available (e.g., MIPS protein complex database for yeast species Pagel et al., 2005 and CORUM protein complex catalog for mammalian species Giurgiu et al., 2019); these can be regarded as prior information and used to enhance the detection of protein complexes for supervised algorithms. Therefore, various supervised models have been devised to identify protein complexes by simultaneously considering both prior knowledge and topological structures of PPI networks. To the best of our knowledge, SCI-BN (Qi et al., 2008) is the first supervised protein complex detection algorithm; this was proposed by using conditional probability for a subgraph of complex building. A Bayesian network was first calculated for each subgraph; the topological and biological features of known complexes were then used to train the BN model to enable estimation of parameters in the model. Finally, the underlying protein complexes in PPI networks were exploited in accordance with confidence scores generated by the trained BN model. Recently, a novel neural network-based semi-supervised model has been developed (Shi et al., 2011), in which a neural network was trained using combinations of both topological and biological features of known protein complexes. In contrast to these two supervised learning models, multiple complex features have been extracted from true protein complexes and subjected to regression analysis to discover protein complexes (Yu et al., 2014). Notably, protein complexes can be obtained from individual experiments (e.g., tandem affinity purification with mass spectrometry), but this process is inefficient because biological experiments are usually time-consuming and expensive (Wang et al., 2019).

The supervised algorithms described above can detect true protein complexes that may be missed by unsupervised algorithms; however, prior knowledge regarding known complexes does not directly enable identification of protein complexes. This may hamper the use of prior information, such that real protein complexes cannot be mined effectively. Most algorithms regard a protein complex as a module that has a set of proteins with similar linkage patterns (Cao et al., 2018). The cluster separation principle (Yu and Xu, 2014) indicates that a good protein complex detection result should have a minimum number of links among modules to imply clear module structure. However, there has been minimal attention to the module structures of protein complexes detected by the supervised and unsupervised algorithms mentioned above.

To address these challenges, we propose a novel semi-supervised protein complex detection model based on non-negative matrix tri-factorization (SSNMTF). This combines network topological structure and prior information indicating that some proteins belong to the same protein complex, thus enabling efficient discovery of protein complexes from PPI networks. Moreover, the physical meanings of the two factorized matrices in SSNMTF are clear, such that one matrix depicts protein community membership and the other matrix indicates module structure. Additionally, the prior information is modeled as a pairwise constraint to guide the learning process regarding each protein's module membership and clarify the module structures of detected protein complexes.

In this work, our main contributions are as follows:

1. We propose a novel semi-supervised module detection model (SSNMTF) that uses protein pairwise constraint as prior information to identify non-overlapping or overlapping protein complex from weighted or un-weighted PPI networks.
2. We use prior information to simultaneously guide the learning processes of a protein membership indicator matrix and a module relationship matrix in SSNMTF.
3. We develop a novel parameter-free method to automatically identify overlapping protein complexes using the protein membership indicator matrix.
4. We provide a corresponding initialization strategy for each respective variable to ensure that the proposed model SSNMTF has a stable solution.

## 2. Materials and Methods

In this section, we provide a detailed demonstration of the proposed novel semi-supervised protein complex detection model based on non-negative matrix tri-factorization. The process of protein complex identification is likely to benefit from incorporating prior information, which is available from various sources. For example, when the gene expression level or semantic similarity and biological meaning are comparable between two proteins, this valuable prior information can support the protein complex detection algorithm by allowing clustering into a single protein complex, although these proteins may not directly interact in a PPI network. Furthermore, we show that the proposed model SSNMTF is more efficient than traditional NMF-based methods for detecting protein complexes.

In this manuscript, we denote a PPI network by an undirected graph *P* = (*V, E*), where $V={\left\{V_{i}\right\}}_{i=1}^{n}$ is a collection of *n* proteins and *E* is a set of *m* edges, such that each edge links a pair of proteins in *V*. Generally, a symmetric adjacency matrix $\text{A}=\left[a_{ij}\right]\in {\mathbb{R}}_{+}^{n\times n}$ is used to indicate an undirected graph *P* and the element *a*_*ij*_ indicates the weight of a link between the *ith* and *jth* proteins. If a PPI network is un-weighted, by convention, we set *a*_*ij*_ = 1 if and only if the *ith* protein and *jth* proteins interact with each other; otherwise, we set *a*_*ij*_ = 0. Because of the limited small amount of protein-protein interaction data, the adjacency matrix **A** is sufficiently sparse to elicit poor performance from protein complex detection algorithms.

### 2.1. Non-negative Matrix Factorization

NMF is a method of decomposing a given original non-negative matrix into low rank non-negative matrices; multiplication of these matrices can approximate the original matrix. The NMF exhibits great power with respect to better parts-of-whole interpretability of the factorized matrices, and it has been successfully applied to a broad range of real-world context (e.g., image, text, information retrieval, community detection, and bioinformatics) (Liu et al., 2017b; Ma et al., 2018). NMF comprises a high-performance approach to detect protein complexes within PPI networks. Non-negative matrix factorization-based models have been designed to identify modules from complex networks; furthermore, NMF-based algorithms have a strong scalability for the use of prior information (Yang et al., 2014). However, the physical meaning of the two factorized matrices is unclear, and the approximating matrix produced by these matrices cannot describe specific links between any two proteins (Binesh and Rezghi, 2018) in PPI networks.

In the traditional NMF model, the Euclidean distance between original matrix and the product of these two factorized matrices is regarded as the cost function, and its objective function is defined as follows.

(1) $$\begin{array}{l} J_{1}\left(W,H\right)=\underset{W\geq 0,H\geq 0}{\min}||A-WH^{T}|{|}_{F}^{2} \end{array}$$

where $\text{W}=\left[w_{ij}\right]\in {\mathbb{R}}_{+}^{n\times k}$ is the base matrix and $\text{H}=\left[h_{ij}\right]\in {\mathbb{R}}_{+}^{n\times k}$ is the module indicator matrix and k (*k* ≪ *n*) is the number of modules. However, the intuitional physical meanings of the two factorized matrices **W** and **H** are ambiguous (Binesh and Rezghi, 2018), and their product cannot effectively describe the original interaction between protein pairs because the rows and columns of matrix **A** are indexed by proteins. However, there is no evidence that the rows and columns of the approximating matrix (produced by matrices **W** and **H**) are indexed by the same objects (Zhu et al., 2007). Because the adjacency matrix **A** is symmetric, the symmetric NMF (SNCF) (Ou-Yang et al., 2013) community detection method has been proposed to uncover protein complexes and the objective function is involved as

(2) $$\begin{array}{l} J_{2}\left(H\right)=\underset{H\geq 0}{\min}||A-HH^{T}|{|}_{F}^{2} \end{array}$$

Notably, the eigenvalues of a semi-positive definite matrix **Ā** = **HH**^**T**^ are all non-negative, but the eigenvalues of the original matrix **A** might be negative. Therefore, SNMF is unsuitable for approximating the original matrix (Zhang and Yeung, 2012). Moreover, the diagonal entities of **Ā** are generally positive, whereas the corresponding entities of matrix **A** are equal to zero.

To overcome the drawback of NMF and SNMF, a bounded nonnegative matrix tri-factorization (Jing et al., 2012), NMTF, has been proposed which is formulated as

(3) $$\begin{array}{l} J_{3}\left(F,G\right)=\underset{F\geq 0,G\geq 0}{\min}||A-FGF^{T}|{|}_{F}^{2} \end{array}$$

where *k* (*k* ≪ *n*) is the number of modules, $\text{F}\in {\mathbb{R}}_{+}^{n\times k}$ is a protein membership indicator matrix, and $\text{G}\in {\mathbb{R}}_{+}^{k\times k}$ is a symmetric module relationship matrix that is responsible for the negative eigenvalues. Furthermore, **G** can be regarded as the structure of detected modules because *g*_*ij*_ describes the relationship between modules *i* and *j*. Additionally, the product of **FGF**^**T**^ can identify relationships between any two proteins. Therefore, NMTF is used in this work to detect protein complexes from PPI networks.

### 2.2. Clear Module Structure With Pairwise Constraint

The pairwise constraint utilized in this work is the must-link constraint that indicates that two proteins should be clustered into the same protein complex (i.e., two corresponding proteins with the must-link constraint should have the same module label). If we know that the prior information which formulated as must-link constraint in advance, then a must-link constraint matrix $\text{M}=\left[m_{ij}\right]\in {\mathbb{R}}_{+}^{n\times n}$ is constructed, where *m*_*ij*_ = α if the *ith* protein and the *jth* protein belong to a same protein complex; otherwise, *m*_*ij*_ = 0. In this work, the must-link constraint is introduced into the NMTF model to simultaneously guide the learning processes of protein membership indicator matrix **F** and module structure matrix **G**.

To ensure that a protein pair (*i* and *j*) with must-link constraint has the same module label, the two corresponding module indicator vectors (**f**_*i*_ and **f**_*j*_) should be similar to each other. Thus, the distance between **f**_*i*_ and **f**_*j*_ should be as small as possible. Euclidean distance is utilized as the distance metric between two vectors in this work which can be denoted as $d_{1}\left({\text{f}}_{i},{\text{f}}_{j}\right)=||{\text{f}}_{i}-{\text{f}}_{j}|{|}_{2}^{2}$. Then the learning process of matrix **F** can be guided by the must-link constraint matrix **M** with the following term:

(4) $$\begin{array}{l} \begin{array}{l} P_{1}\left(F\right)=\underset{F}{\min}\frac{1}{2}\times \underset{i,j}{\sum }m_{i,j}\times d_{1}\left({\text{f}}_{i},{\text{f}}_{j}\right)\\ \;=\underset{F}{\min}Tr\left(F^{T}DF\right)-Tr\left(F^{T}MF\right)\\ \;=\underset{F}{\min}Tr\left(F^{T}LF\right) \end{array} \end{array}$$

where $\text{D}=\left[d_{i,j}\right]\in {\mathbb{R}}_{+}^{n\times n}$ is the diagonal matrix for matrix **M** ($d_{i,i}=\sum_{j=1}^{n}m_{i,j}$) and **L** = **D** − **M** is the Laplacian matrix, *Tr*(•) denotes the trace of matrix. In each row of matrix **F**, the index of the largest element is the protein complex label for the corresponding protein.

**Figure 1:**
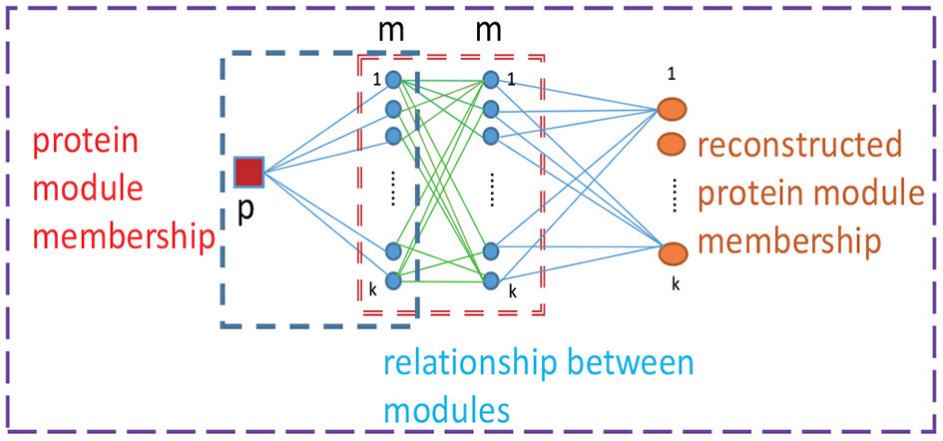
Reconstruction of propensity between a protein and modules. p denotes a protein, m represents a module and k represents the number of modules.

It is difficult to use prior information to guide the learning process regarding the relationship of the detected protein complexes to directly obtain a clear module structure. Moreover, we noticed that matrix **G** depends on matrix **F**, thus, the presence of matrix **F** implies a corresponding matrix **G**. The (*i, c*)*th* element *f*_*ic*_ denotes the propensity that the *ith* protein belongs to the *cth* module. Considering the relationships between the *cth* module and all detected modules, we reconstruct a new propensity that the *ith* protein belongs to the *cth* module by $r_{ic}=\underset{k}{\sum }f_{ik}g_{kc}$. For example, as shown in Figure 1, the reconstructed propensity between protein *p* and module 1 can be acquired by $r_{p1}=\underset{k}{\sum }f_{pk}g_{k1}$. Accordingly, when we reconstruct the probability that protein *p* belongs to module 1, the propensities between protein *p* and all detected modules are considered, as are the relationships between module 1 and all modules.

The reconstructed matrix is defined as $\text{R}=\left[r_{ic}\right]\in R_{+}^{n\times k}=\text{F}\text{G}$, which can be viewed as a new membership matrix for all proteins. From a mathematical perspective, with the aid of module structure matrix **G**, we projected the protein membership indicator matrix **F** into the protein-to-module relationship matrix **R**; each row of **R** denotes the reconstructed protein module indicator vector for each protein. The two corresponding rows (*r*_*i*_ and *r*_*j*_) of a protein pair (*i* and *j*) with the must-link constraint should also be similar to each other to enable clustering into a single protein complex. Hence, to achieve a clear module structure of all detected protein complexes, we proposed a novel penalty term for the must-link constraint. The Euclidean distance is used to measure the difference between **r**_*i*_ and **r**_*j*_ which denotes the memberships for the *ith* and *jth* proteins, respectively. Thus, **r**_*i*_ and **r**_*j*_ should be as close to each other as possible if proteins *i* and *j* with the must-link constraint. This yield $d_{2}\left({\text{r}}_{i},{\text{r}}_{j}\right)=||{\text{r}}_{i}-{\text{r}}_{j}|{|}_{2}^{2}=||{\left(\text{F}\text{G}\right)}_{i}-{\left(\text{F}\text{G}\right)}_{j}|{|}_{2}^{2}$. Consequently, the novel penalty term of the must-link constraint that is used to achieve a clear module structure can be formulated as follows:

(5) $$\begin{array}{l} \begin{array}{l} P_{2}\left(F,G\right)=\underset{F,G}{\min}\frac{1}{2}\times \underset{i,j}{\sum }m_{i,j}\times d_{2}\left({\text{r}}_{i},{\text{r}}_{j}\right)\\ \;=\underset{F,G}{\min}Tr\left({\left(FG\right)}^{T}D\left(FG\right)\right)-Tr\left({\left(FG\right)}^{T}M\left(FG\right)\right)\\ \;=\underset{F,G}{\min}Tr\left({\left(FG\right)}^{T}L\left(FG\right)\right) \end{array} \end{array}$$

where the matrices **D**, **M**, and **L** are the same as in Equation (4). Moreover, this term can be utilized to concurrently guide the learning process of matrices **F** and **G**.

### 2.3. SSNMTF

The main idea in this work is not to simply incorporate the prior information into the original PPI network;instead, it is intended to merge prior information into a unitary model to concurrently guide the learning process of protein membership indicator matrix **F** and module structure matrix **G**. Therefore, the objective function of the proposed model SSNMTF has been formulated as follows:

(6) $$\begin{array}{l} \begin{array}{l} J\left(F,G\right)=\underset{F\geq 0,G\geq 0}{\min}||A-FGF^{T}|{|}_{F}^{2}+Tr\left({\left(FG\right)}^{T}L\left(FG\right)\right)\\ \;+Tr\left(F^{T}LF\right) \end{array} \end{array}$$

The second term is used to learn a more rational **G**, thus ensuring that the module structure of the detected protein complexes is clear. The corresponding protein complexes can more easily be separated from each other. The third term is used to learn the protein membership indicator matrix, which can ensure that two proteins with the must-link constraint are clustered into a single protein complex. According to the trace knowledge of matrices such as *Tr*(*A*) = *Tr*(*A*^*T*^), $||A|{|}_{F}^{2}=Tr\left(AA^{T}\right)$ and *Tr*(*AB*) = *Tr*(*BA*), Equation (6) can be rewritten as follows:

(7) $$\begin{array}{l} \begin{array}{l} J\left(F,G\right)=\underset{F\geq 0,G\geq 0}{\min}Tr(FGF^{T}FGF^{T}-AFG^{T}F^{T}\\ \;-FGF^{T}A^{T}+AA^{T})+Tr\left({\left(FG\right)}^{T}L\left(FG\right)\right)\\ \;+Tr\left(F^{T}LF\right) \end{array} \end{array}$$

To satisfy the non-negative constraints on matrices **F** = [*f*_*ij*_] ≥ 0 and **G** = [*g*_*ij*_] ≥ 0, two Lagrange multipliers $\Psi =\left[\psi_{ij}\right]\in {\mathbb{R}}_{+}^{n\times k}$ and $\Phi =\left[\phi_{ij}\right]\in {\mathbb{R}}_{+}^{k\times k}$ are introduced for each respective matrix. Then the lagrange (Equation 7) can be rewritten as follows:

(8) $$\begin{array}{l} \begin{array}{l} J_{l}\left(F,G\right)=\underset{F,G}{\min}Tr(FGF^{T}FGF^{T}-AFG^{T}F^{T}-FGF^{T}A^{T}\\ \;+AA^{T})+Tr\left({\left(FG\right)}^{T}L\left(FG\right)\right)+Tr\left(F^{T}LF\right)\\ \;+Tr\left(\text{Ψ}F^{T}\right)+Tr\left(\Phi G^{T}\right) \end{array} \end{array}$$

Equation (8) is not convex with both matrices **F** and **G** as concurrent variables, but it is convex when one matrix (only **F** or only **G**) is constant. To obtain optimal **F** and **G**, thus achieving a local minimum for (Equation 6), we iteratively update one matrix while keeping the other fixed. The partial derivatives of Equation (8 against variable matrices **F** and **G** are

(9) $$\begin{array}{l} \begin{array}{l} \frac{\partial J_{l}}{\partial F}=2FGF^{T}FG-2AFG+LFGG+LF+\text{Ψ}\\ \frac{\partial J_{l}}{\partial G}=F^{T}FGF^{T}F-F^{T}AF+GF^{T}LF+\Phi \end{array} \end{array}$$

By letting the partial derivatives of **F** and **G** be equal to zero and employing Karush-Kuhn-Tucker (KKT) conditions ψ_*ik*_*f*_*ik*_ = 0 and ϕ_*jk*_*g*_*jk*_ = 0, then the updating rules of matrices **F** and **G** can be obtained as follows:

(10) $$\begin{array}{l} F=F⊗\frac{2AFG+MF\left(GG+I\right)}{2FGF^{T}FG+DF\left(GG+I\right)} \end{array}$$

(11) $$\begin{array}{l} G=G⊗\frac{F^{T}AF+GF^{T}MF}{F^{T}FGF^{T}F+GF^{T}DF} \end{array}$$

where ⊗ represents the Hadamard product of two matrices, **I** is the identity matrix. The two matrices **F** and **G** will be updated iteratively until the value of Equation (6) does not change or the maximum number of iterations set in advance has been reached.

### 2.4. Initialization Strategy

Because the proposed model SSNMTF is sensitive to initial values of matrices **F** and **G**, we provide an initialization strategy for each matrix, thus achieving a stable solution for SSNMTF. A community detection algorithm named K-rank-D (Li et al., 2015) has been proposed in which initial seeds for all modules are provided. The meaning of matrix **C**, composed of these seeds, is similar to the meaning of matrix **F** in SSNMTF. Therefore, we initially set **F** = **C**. Because **G** describes the module structure of predicted protein complexes, we initially expect that the relationships are similar among all protein complexes. The following two strategies are then used to initialize **G** in a row specific manner. For the *ith* row, the sum of all elements is one, $\sum_{j=1}^{k}g_{ij}=1$. Moreover, the value of each element is close to but not equal to 1/*k*, *g*_*i, j*_ = 1/*k* + ϵ, where ϵ is a small value and *k* is the number of predicted protein complexes. We describe the proposed SSNMTF model in Algorithm 1, ϵ was set to 10^−15^ and the maximum number of iterations τ_*max*_ was set to 1,000 in this work.

**Algorithm 1:** The proposed SSNMTF

**Input:** parameter α, adjacency matrix *A* ∈ *R*^*n*×*n*^, must-link set MS, number of protein complexes *k*, maximum number of iterations τ_*max*_
1: Construct the must-link constraint matrix *M* ∈ *R*^*n*×*n*^ in terms of MS
2: **Initialize:** *F* and *G* according to the initialization strategy, t = 1
3: **while** not converge **do**
4: Keep matrix *G* fixed, and update matrix *F* according to Equation (10)
5: Keep matrix *F* fixed, and update matrix *G* according to Equation (11)
6: Calculate the value *J*_*t*_ of objective fuction according to Equation (6)
7: Check the convergence: if $\frac{\left|J_{t+1}-J_{t}\right|}{J_{t}}<\epsilon$ or *t* > τ_*max*_, else t = t + 1
8: **end while**
**Output:** module indicator matrix F and module relationship matrix G

### 2.5. Protein Complex Detection

#### 2.5.1. Non-overlapping Protein Complex Detection

The element *f*_*ic*_ in matrix **F** indicates the strength of the prediction that protein *i* belongs to protein complex *c*. Accordingly, the index *c* with the maximum value in the *ith* row is the assigned protein complex of protein *i*, which can be expressed as $c=arg\underset{c}{\text{ max }}f_{ic}$. The number of protein complexes is difficult to determine because prior information is sparse regarding the number of modules in real PPI networks. Each column in **F** may represent an empty module if no value is the largest value in its corresponding row. Therefore, *k* can receive a comparatively large value to ensure that it can adaptively determine the number of modules. In this work, the proposed non-overlapping protein complex detection method is denoted as NSSNMTF.

#### 2.5.2. Overlapping Protein Complex Detection

We propose a novel parameter-free method to detect overlapping protein complexes, using matrix **F**. Suppose that we have four proteins *p*_1_, *p*_2_, *p*_3_, *p*_4_ that belong to three protein complexes *m*_1_, *m*_2_, *m*_3_, where *m*_1_ = {*p*_1_, *p*_2_}, *m*_2_ = {*p*_2_, *p*_3_, *p*_4_} and *m*_3_ = {*p*_1_, *p*_4_}. The protein membership indicator matrix **F** can be constructed for all proteins, such that *f*_*ij*_ = 1 if protein *i* belongs to protein complex *j*, otherwise, *f*_*ij*_ = 0. Notice that the values of the product of **F**^**T**^**F** provide the overlap (number of member proteins) of corresponding protein complexes, accordingly, the diagonal elements indicate the size of corresponding protein complexes (these diagonal elements will be real values when the value of the element in **F** ranges from 0 to 1).

(12) $$\begin{array}{l} F=\left[\begin{array}{ccc} 1 & 0 & 1\\ 1 & 1 & 0\\ 0 & 1 & 0\\ 0 & 1 & 1 \end{array}\right]F^{T}F=\left[\begin{array}{ccc} 2 & 1 & 1\\ 1 & 3 & 1\\ 1 & 1 & 2 \end{array}\right] \end{array}$$

In PPI network applications, each column of **F** represents a protein complex and each entry of one column indicates the strength of a protein's contribution to this protein complex. Therefore, all entries are ranked in descending order according to their values in each column. For any protein complex *c*, the *cth* column of ordered **F** is indicated by ${\hat{f_{c}}}^{T}=\left(\hat{f_{1}^{c}},\hat{f_{2}^{c}},\ldots ,\hat{f_{n}^{c}}\right)$, where $\hat{f_{1}^{c}}>\hat{f_{2}^{c}}>\ldots >\hat{f_{n}^{c}}$. Thus, the corresponding protein vector of ${\hat{f_{c}}}^{T}$ is expressed by $p_{c}^{T}=\left(p_{1}^{c},p_{2}^{c},\ldots ,p_{n}^{c}\right)$ where $p_{i}^{c}$ is the protein associated with value $\hat{f_{i}^{c}}$. We sequentially accumulate the elements in $\hat{f_{c}}$ until their sum is greater than or equal to the size of module *c* which is the *cth* item in a vector composed of the diagonal elements of matrix **F**^**T**^**F**. For example, if $\hat{f_{1}^{c}}+\hat{f_{2}^{c}}+\hat{f_{2}^{c}}\geq {\left(F^{T}F\right)}_{cc}$ and $\hat{f_{1}^{c}}+\hat{f_{2}^{c}}<{\left(F^{T}F\right)}_{cc}$, the corresponding proteins $p_{1}^{c}$, $p_{2}^{c}$ and $p_{3}^{c}$ will be selected as members of the *cth* protein complex. The detected protein complexes will overlap with each other if they are essentially overlapped. The predicted protein complexes containing fewer than three member proteins will be filtered out. We also assign *k* a comparatively large value, which allows it to adaptively determine the number of modules. In this manuscript, the proposed overlapping protein complex detection method is denoted as OSSNMTF.

### 2.6. Datasets

In this manuscript, five binary PPI networks of humans extracted from HuRI (Luck et al., 2020), MINT (Licata et al., 2012), BioGRID (Oughtred et al., 2019), mentha (Calderone et al., 2013), and STRING (version 11) (Szklarczyk et al., 2019), respectively, and one weighted PPI network extracted from STRING are used. The network we extracted from BioGRID is based on BioGRID multi-validated datasets in which each physical interaction passes a specific set of criteria. Each interaction between a protein pair in STRING has a confidence score. We extract a weighted PPI network named WSTRING, in which the confidence score of each interaction is ≥700 (Ananthasubramanian et al., 2012). In addition, an un-weighted PPI network named STRING is extracted from WSTRING by ignoring the weights of interactions.

Two manually curated protein complex databases are used as references. The first is the human protein complex database with a complex quality index (PCDq) (Kikugawa et al., 2012), which includes both known and predicted complexes. The second protein complex database is extracted from CORUM (Giurgiu et al., 2019), including a set of manually annotated mammalian protein complexes, by filtering out nonhuman protein complexes. Additionally, the prior information is derived from CORUM and PCDq (details are provided in section D). Proteins absent from the corresponding PPI networks are filtered out from PCDq and CORUM, respectively. Moreover, we only retained protein complexes comprising at least three distinct proteins. The detailed information regarding the original PPI networks and corresponding protein complex databases is shown in Table 1,

**Table 1:** Information of 5 human PPI networks and 2 golden standard complex databases.

**PPI network**	**#protein**	**#edge**	**CORUM**			**PCDq**		
			***#cc***	**#cp**	**#as**	**#cc**	**#cp**	**#as**
HuRI	8,135	52,398	259	840	6.05	322	1,046	4.17
BioGRID	8,766	4,0621	762	2,012	6.31	725	2,473	4.63
MINT	9,995	31,324	674	1,763	6.21	618	2,088	4.59
mentha	16,584	180,948	815	2,139	6.15	890	2,974	4.41
STRING	17,185	420,534	821	2,143	6.17	881	2,989	4.51

#protein is the number of proteins and #edge is the number of interactions in PPI networks. #cc and #cp denote the respective numbers of covered complexes and proteins for each human PPI network according to different complex references, and #as represents the mean size of the covered complexes. The information regarding WSTRING is identical to the information describing STRING.

### 2.7. Evaluation Metrics

To evaluate the performance for protein complex detection algorithms, the metrics such as cluster-wise Sensitivity (Sn), cluster-wise positive predictive value (PPV), Accuracy (Acc), precision, recall, F1 (Cao et al., 2016) and maximum matching ratio (MMR) (Nepusz et al., 2012) are used to assess the similarity between the predicted protein complexes (*P*) and the golden standard complex sets (*G*). To measure the matching rate between a predicted protein complex and a benchmark one, we follow the neighborhood affinity (NA) score which is defined as follows:

(13) $$\begin{array}{l} NA\left(p,g\right)=\frac{|N_{p}\cap N_{g}{|}^{2}}{\left|N_{p}||N_{g}\right|} \end{array}$$

NA is used to measure the overlapping degree between a predicted protein complex *p* and a golden standard reference complex *g*, and *p*, and *g* are regarded to match each other when *NA*(*p, g*) ≥ 0.25. Then the precision, recall, F1 sensitivity (Sn), positive predictive value (PPV), and accuracy (Acc) are introduced to assess the performance of the protein complex detected algorithms. After all detected protein complexes get their best matched real protein complexes in terms of NA values, then precision, recall, and F1 are defined as

(14) $$\begin{array}{l} \begin{array}{c} N_{cb}=|\left\{b|b\in G_{c},\exists p\in D_{s},OL\left(p,b\right)\geq 0.2\right\}|\\ N_{cp}=|\left\{p|p\in D_{s},\exists b\in G_{c},OL\left(b,p\right)\geq 0.2\right\}| \end{array} \end{array}$$

(15) $$\begin{array}{l} Precision=\frac{\left|N_{cp}\right|}{\left|D_{s}\right|},Recall=\frac{\left|N_{cb}\right|}{\left|G_{c}\right|} \end{array}$$

(16) $$\begin{array}{l} F1=\frac{2\times Precision\times Recall}{Precision+Recall} \end{array}$$

Let *m* = |*P*|, *n* = |*G*|, *t*_*ij*_ denote the number of common proteins that exist in both the *ith* predicted protein complex and *jth* reference golden standard complex, and *N*_*j*_ denote the number of proteins in the *jth* complex and then Sn, PPV and Acc are defined as follows:

(17) $$\begin{array}{l} Sn=\frac{\sum_{j=1}^{n}max_{i}\left\{t_{ij}\right\}}{\sum_{j=1}^{n}N_{j}} \end{array}$$

(18) $$\begin{array}{l} PPV=\frac{\sum_{i=1}^{m}max_{j}\left\{t_{ij}\right\}}{\sum_{i=1}^{m}\sum_{j=1}^{n}t_{ij}} \end{array}$$

(19) $$\begin{array}{l} Acc=\sqrt{Sn\times PPV} \end{array}$$

Sn is used to measure the proteins in predicted protein complexes covered by reference complexes, PPV is utilized to assess the matching rate between detected protein complexes and golden standard complexes and acc is the geometric means of Sn and PPV. MMR is calculated by a maximal one-to-one mapping between predicted protein complexes and gold standard complexes which is defined as follows:

(20) $$\begin{array}{l} MMR=\frac{\sum_{i=1}^{n}max_{j}NA\left(i,j\right)}{n} \end{array}$$

The enrichment analysis for predicted protein complexes was performed by calculating the *p*-value from the hypergeometric distribution (Li et al., 2010), which is defined as follows:

(21) $$p-value=\sum_{x=q}^{f}\frac{\left(\begin{array}{l} f\\ x \end{array}\right)\left(\begin{array}{l} t-f\\ t-x \end{array}\right)}{\left(\begin{array}{l} t\\ k \end{array}\right)}$$

where *t* represents the total number of proteins in the PPI network, *k* is the number of proteins in one identified protein complex, *f* denotes the number of proteins annotated by any one Gene Ontology (GO) term *g*_*t*_ and *q* indicates the number of proteins annotated by *g*_*t*_ in any one detected protein complex. The smaller the *p*-value, the more biologically significant the corresponding detected protein complex (Bhowmick and Seah, 2015), in other words, a predicted protein complex with a smaller *p*-value is more likely to be a true protein complex.

## 3. Results

We performed experiments regarding both synthetic benchmark networks and human-related PPI networks to investigate the effectiveness of our proposed models NSSNMTF and OSSNMTF. We first assessed the performances of NSSNMTF and OSSNMTF on synthetic networks with known benchmarks. The LFR network generator was used to produce networks with non-overlapping and overlapping modules. To clarify the effectiveness of our proposed models NSSNMTF and OSSNMTF, we compared the following algorithms: six semi-supervised module detection algorithms (i.e., PCNMF, PCSC Yang et al., 2014, CPSNMF Wang et al., 2015, PCNMTF Liu et al., 2017a, svdcnmf Lu et al., 2020, and SNFM Man et al., 2019, which can use identical pairwise constraints and a single initial protein membership matrix **F**, as in our proposed algorithms), and two unsupervised module identification algorithms (i.e., ClusterONE Nepusz et al., 2012 and NCMine Tadaka and Kinoshita, 2016, which can identify overlapping protein complexes). All parameters used in each compared algorithm were set according to the suggestions of their authors. Additionally, PCNMF, CPSNMF, PCSC, svdcnmf, SNFM, PCNMTF, and ClusterONE have been shown to identify protein complexes from weighted PPI networks.

### 3.1. Parameter Analysis

**Figure 2:**
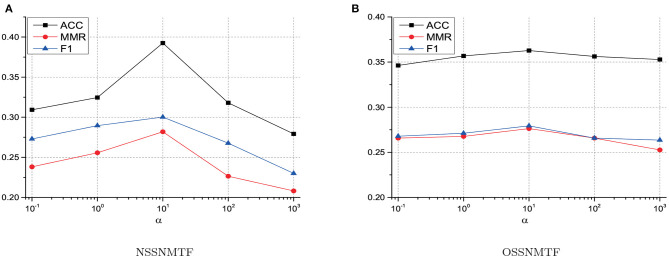
The impact of parameter α on the performance of NSSNMTF and OSSNMTF. **(A)** The performance of NSSNMTF with different values of α on HuRI. **(B)** The performance of OSSNMTF with different values of α on HuRI. The *x*-axis represents the value of α, *y*-axis denotes values of ACC, MMR and F1.

Only one parameter α used in SSNMTF can balance the tradeoff between topological information and priori information. To explore how this parameter affects the performance of SSNMTF, we evaluated the performance of our proposed two algorithms NSSNMTF and OSSNMTF on HuRI network as α varied from 0.1 to 1,000. To discuss how to determine α, for better illustration, we displayed ACC, MMR, and F1 (mean values over 50 experiments) with different α by taking CORUM as the golden standard in Figure 2. Since the must-link information used in this work comes from the golden standard protein complexes, the performance of our proposed algorithms consistently increases as we weight more on must-link information. Figures 2A,B show that as the value of α increases, the performances of NSSNMTF and OSSNMTF improve at first and then decrease after α is larger than 10. Taking NSSNMTF on HuRI network as an example due to the fact that NSSNMTF is more sensitive than OSSNMTF as shown in Figure 2, as 10% must-link is used, the ACC reaches 0.31, 0.32, and 0.39 when we set α as 0.1, 1, and 10 respectively. However, the ACC reaches 0.32 and 0.28 when we set α as 100 and 1,000. The reason is that according to the objective function of the proposed mode SSNMTF (Equation 6), α controls the contribution of the topological structure of the PPI network and must-link information. At the beginning, increasing α can significantly improve the performance (e.g., α = 10) which means the must-link information plays a important role in detecting protein complexes, but the performance degrades when we set a large α (e.g., α = 1,000) that indicates (Equation 6) pays much more attention to must-link information than to the topological structure of a PPI network. From Figure 2, we can find that both NSSNMTF and OSSNMTF perform better when α is in the vicinity of 10 in terms of ACC, MMR, and F1. The influences of α on other human PPI networks are similar to the influences on HuRI, thus the only one parameter α in SSNMTF is set to 10 for all experiments.

### 3.2. Synthetic Networks

To clarify the performance difference of all compared methods, we first evaluated the performance improvement on synthetic networks. The LFR networks (Lancichinetti et al., 2008) generator can produce networks with known overlapping and non-overlapping ground truth and it allows to specify the generated networks with serval parameters including *N* (number of nodes), *ad* (average degree of nodes), *d*_*max*_ (maximum degree of nodes), *m*_*min*_ (minimum module size), *m*_*max*_ (maximum module size), μ (mixing parameter), *on* (number of overlapping nodes), and *om* (number of modules for overlapping nodes). The mixing parameter μ determines the clarity of the topological structure, and the greater value of μ means the more blurred module structure of generated networks. In this manuscript, we set *N* = 1, 000, *ad* = 15, *d*_*max*_ = 50, *m*_*min*_ = 20, *m*_*max*_ = 50 and μ varying from 0.6 to 0.7 for non-overlapping and overlapping module benchmark networks. In addition, for overlapping module benchmark networks, we set *on* = 200 and *om* = 2. The normalized mutual information (NMI) (Lancichinetti et al., 2009) was used to evaluate performances for both non-overlapping and overlapping module detection methods. A larger value of NMI is associated with algorithm based detection of a better module result.

**Figure 3:**
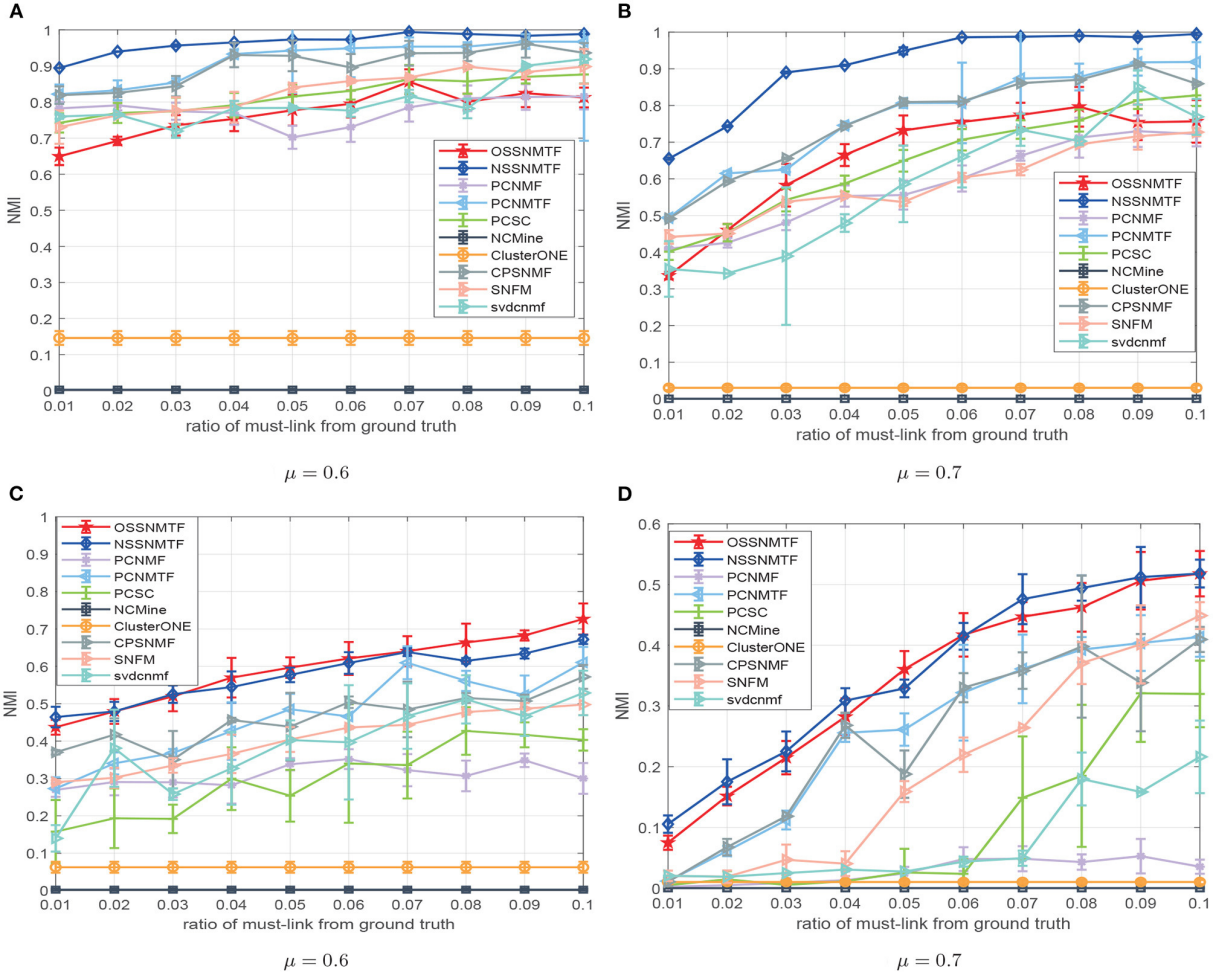
NMI of compared methods with respect to diverse percentages of must-link constraints extracted from ground-truth. **(A,B)** Regarding LFR networks with non-overlapping modules; **(C,D)** Regarding LFR networks with overlapping networks. The *x*-axis represents the ratio of must-link constraints, *y*-axis denotes value of NMI.

The must-link constraints were derived from ground-truth with the same method as was introduced in Yang's work (Yang et al., 2014). We generated 100 LFR networks in a random manner, using the parameters introduced above for different μ; we then reported the mean NMI and standard deviation in terms of different percentages of must-link constraints in Figure 3. With respect to non-overlapping (Figures 3A,B) and overlapping (Figures 3C,D) network benchmarks, NSSNMTF is superior to PCNMF, PCSNMF, and PCSC, while OSSNMTF is superior to ClusterONE and NCMine particularly when the modular structure becomes unclear (i.e., a lager μ). Figure 3 shows that prior information regarding must-link constraints can significantly improve the performance of module detection methods. Moreover, the performances of both NSSNMTF and OSSNMTF shows greater increases in ambiguous networks (i.e., μ = 0.7). For example, the NMI of NSSNMTF reached 0.99 when a 7% must-link constraint was used; the second highest NMI 0.87 was observed on non-overlapping networks with μ = 0.7. When the network module structure was ambiguous (Figure 3D) and nodes could belong to multiple modules, the performances of NSSNMTF and OSSNMTF were significantly better than the performances of other compared algorithms.

**Figure 4:**
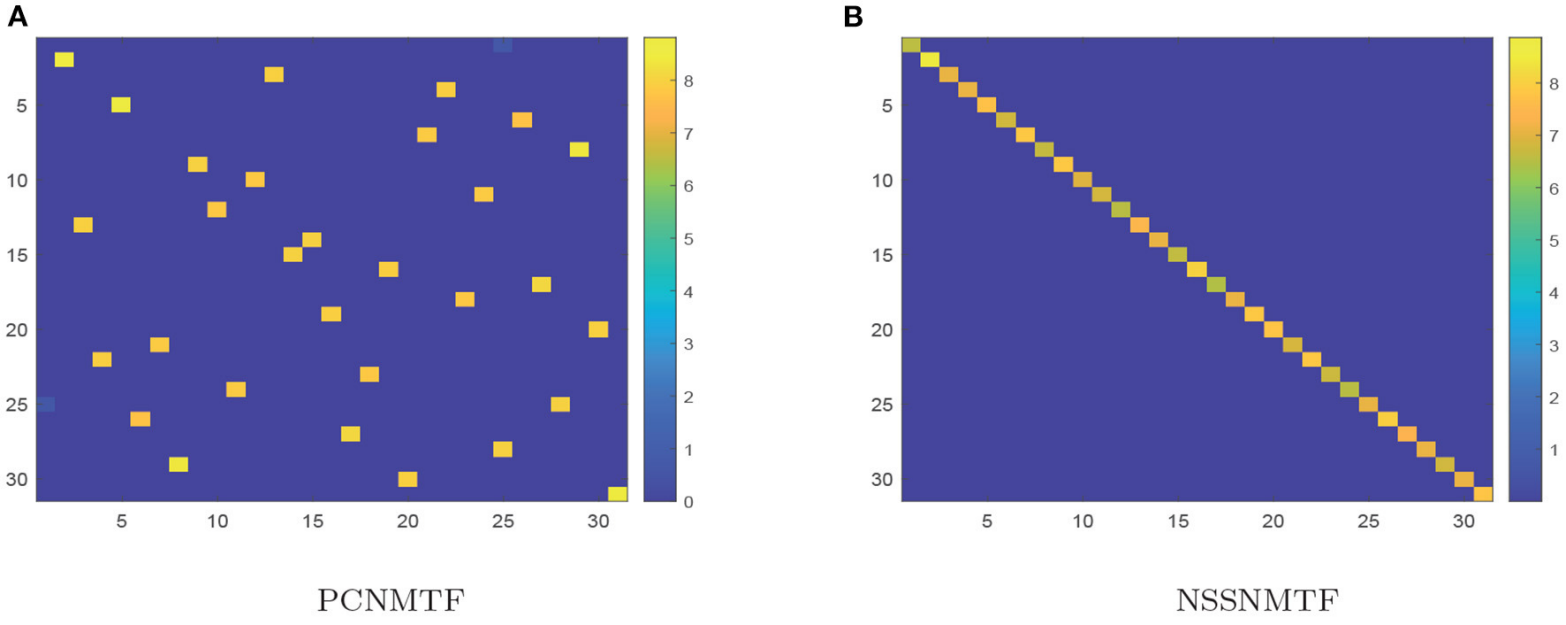
Module structures (matrix **G**) of the LFR (μ = 0.7) network generated by PCNMTF and NSSNMTF. **(A)** Module structure generated by PCNMTF using 1% prior information; **(B)** Module structure predicted by NSSNMTF using 1% prior information. The *x*- and *y*-axis denotes the ID of detected modules. The color scale represents the strength of the correlation between two modules, blue means the correlation is weak, while yellow means strong.

The proposed model SSNMTF outperforms other state-of-the-art module identification approaches on diverse synthetic networks because it appeared to more efficiently make full use of prior information. The pairwise constraint is used to guide the process of leaning module membership for each node; it can also clarify the module structure of detected modules. For example, the module structures of distinct module results generated by PCNMTF and NSSNMTF, using identical prior information, on the LFR network with μ = 0.7 are shown in Figure 4. The relationships among modules detected by PCNMTF are shown in Figure 4A. The findings show that some relationships between predicted modules are larger than the module itself which implies an ambiguous module structure. Furthermore, the module structure matrix **G** is learned by the proposed model NSSNMTF using only 1% pairwise constraint is showed in Figure 4B, the result indicates that the diagonal values are larger than other values, suggesting that the detected modules have a clear module structure.

### 3.3. Human PPI Networks

The prior information (i.e., must-link constraints) used in SSNMTF is extracted from CORUM and PCDq using the approach suggested in Yang et al. (2014). Because some proteins participate in multiple complexes, a must-link constraint is constructed for a protein pair if the two corresponding proteins are involved in only a single protein complex. Thus, *N*_*p*_ = *N*_*c*_(*N*_*c*_ − 1)/2 must-link constraints will be extracted from a specific complex, which contains *N*_*c*_ proteins. In this work, we randomly chose 10% must-link constraints, based on *N*_*p*_, as prior information. The information regarding extracted must-link constraints from CORUM and PCDq for all three human PPI networks is listed in Table 2. To assess the quality of the proposed protein complex detection model SSNMTF, we first evaluated the ability of the proposed model SSNMTF to predict true protein complexes. The initial possible number of detected protein complexes was set to 1,000 for all PPI networks. Although the must-link constraints used as prior information were derived from CORUM and PCDq, these data do not explicitly indicate the protein memberships of protein pairs. Furthermore, the data do not indicate the number of protein complexes in which PPI networks should be clustered. Importantly, the proteins used in must-link constraints are only a subset of data from CORUM and PCDq, and both CORUM and PCDq are used as complex references.

**Table 2:** Information of must-link constraints (10%) for all PPI networks.

**Network**	**#must-link**	**#covered protein**
HuRI	788	1,017
BioGRID	1,249	1,509
MINT	1,352	1,971
mentha	2,569	2,765
STRING	2,563	2,769

**Figure 5:**
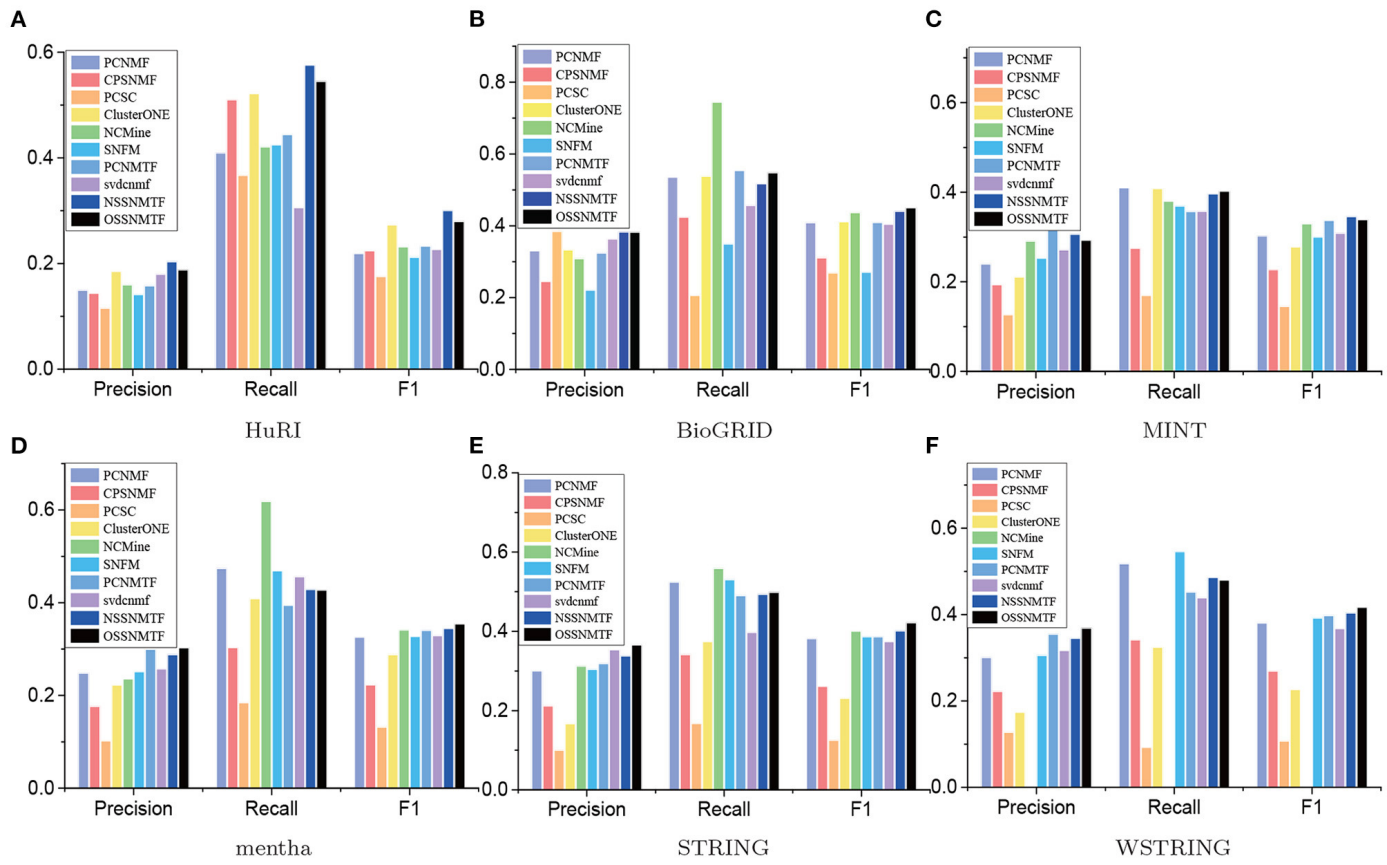
Precision, recall and F1 of compared algorithms on different PPI networks using CORUM as gold standard dataset. The *x*-axis represents precision, recall, and F1, *y*-axis denotes the corresponding values. **(A)** HuRI; **(B)** BioGRID; **(C)** MINT; **(D)** mentha; **(E)** STRING; **(F)** WSTRING.

**Figure 6:**
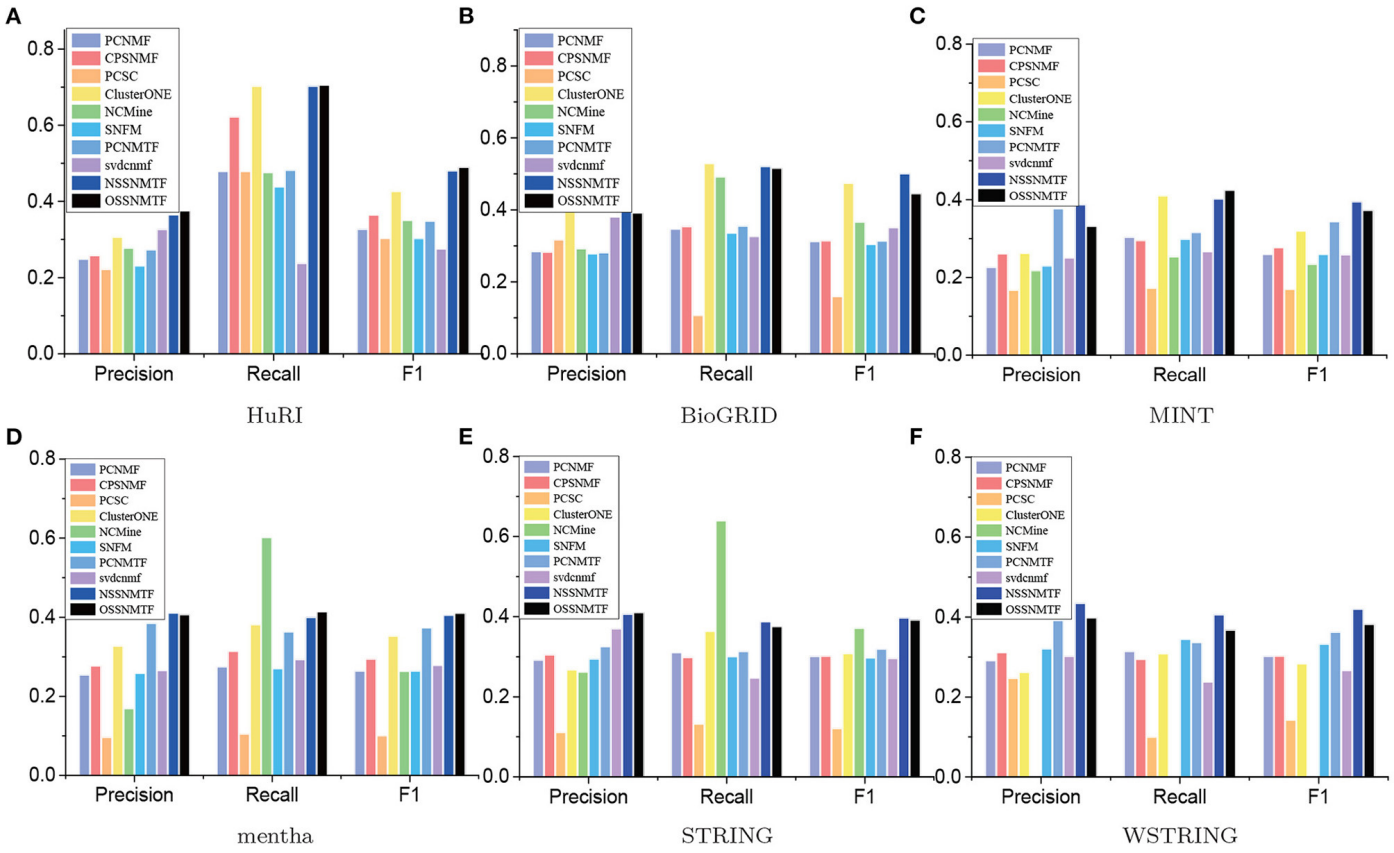
Precision, recall, and F1 of compared algorithms on different PPI networks using PCDq as the gold standard dataset. The *x*-axis represents precision, recall, and F1, the *y*-axis denotes the corresponding values. **(A)** HuRI; **(B)** BioGRID; **(C)** MINT; **(D)** mentha; **(E)** STRING; **(F)** WSTRING.

We first used precision, recall, and F1 as the evaluated metrics to assess the performance of our proposed models NSSNMTF, OSSNMTF and other compared algorithms. The comprehensive comparison results using CORUM and PCDq as gold standard datasets of the five unweighted and one weighted PPI networks are showed in Figures 5, 6, respectively. The proposed models NSSNMTF and OSSNMTF could detect protein complexes with greater accuracy compared with other algorithms. Figures 5A, 6A show that when HuRI was used as the input PPI network, NSSNMTF and OSSNMTF had the highest and second highest F1 values, significantly outperforming other compared algorithms. Regardless of the reference dataset, the performances of our proposed models NSSNMTF and OSSNMTF were superior to the performances of other semi-supervised algorithms that used identical prior information. This included a comparison with the algorithm PCNMTF, which used tri-factorization and prior information in a manner identical to our proposed model, but without considering the module structure. This finding implied that the use of prior information to ensure that identified protein complexes have a clear module structure can markedly improve the accuracy of protein complex detection.

After that, we then employ Sn, PPV, ACC, and MMR as evaluate metrics to compare detected protein complexes with two golden standard datasets. The results of all compared algorithms are listed in Table 3. For each compared method, we demonstrated the number of detected protein complexes (#m) and the number of protein complexes matched with at least one known complex (#mm). For non-overlapping protein complex detection methods, the proposed model NSSNMTF has the highest ACC and MMR scores, compared with other semi-supervised non-overlapping methods. For overlapping protein complex detection methods, the proposed model OSSNMTF outperformed all other overlapping algorithms. The NSSNMTF and OSSNMTF showed the highest and second highest ACC and MMR scores on all five unweighted and one weighted human PPI networks. For example, the highest MMR scores of OSSNMTF were 0.2750 and 0.2434 using CORUM and PCDq as respective golden standards on the STRING network. Additionally, the highest MMR scores of OSSNMTF were 0.2723 and 0.2402 using CORUM and PCDq as respective golden standards, thus indicating that OSSNMTF can achieve better maximal one-to-one mapping to real protein complexes. These results imply that the proposed models NSSNMTF and OSSNMTF can provide a more effective method to detect protein complexes from human PPI networks. Additionally, the ACC and MMR scores of both NSSNMTF and OSSNMTF for weighted PPI networks were higher than those scores for unweighted PPI networks, which suggests that the quality of detected protein complexes can be substantially improved by considering the weights of interactions between protein pairs. Furthermore, the weights of interactions can reduce the impact of noisiness (e.g., false positive interactions) in some PPI networks (Bhowmick and Seah, 2015).

**Table 3:** Detailed results of compared algorithms on six human PPI networks using CORUM and PCDq as gold standards.

**Network**	**Method**	**#m**	**CORUM**					**PCDq**				
			**#mm**	**Sn**	**PPV**	**ACC**	**MMR**	**#mm**	**Sn**	**PPV**	**ACC**	**MMR**
HURI	PCNMF	758	113	0.1971	0.3184	0.2505	0.2131	188	0.2740	0.4178	0.3383	0.2277
	CPSNMF	948	136	0.2602	0.3599	0.3060	0.2436	244	0.3812	0.4761	0.4260	0.2825
	PCSC	782	90	0.2098	0.2726	0.2392	0.1965	173	0.2614	0.3473	0.3013	0.2186
	ClusterONE	1,250	231	0.2883	0.3537	0.3193	0.2751	382	0.3946	0.5022	0.4452	0.3128
	NCMine	2,680	427	0.1658	0.3704	0.2478	0.1887	742	0.2405	0.3664	0.2969	0.2067
	SNFM	745	105	0.1990	0.3111	0.2488	0.2180	172	0.2800	0.4243	0.3447	0.2234
	PCNMTF	730	115	0.2423	0.3373	0.2859	0.2344	199	0.3120	0.4462	0.3731	0.2420
	svdcnmf	613	110	**0.5834**	0.2212	0.3593	0.2220	200	**0.5225**	0.2633	0.3709	0.2217
	NSSNMTF	719	146	0.4158	**0.3706**	**0.3925**	**0.2819**	262	0.4840	**0.5047**	**0.4942**	0.3359
	OSSNMTF	921	173	0.3705	0.3551	0.3627	0.2763	345	0.4698	0.4895	0.4796	**0.3478**
BioGRID	PCNMF	843	278	0.3526	**0.3250**	0.3385	0.2739	239	0.3551	0.3855	0.3700	0.2140
	CPSNMF	894	219	0.4552	0.2233	0.3188	0.2188	252	0.4237	0.2964	0.3544	0.2115
	PCSC	237	91	0.3609	0.2186	0.2809	0.1441	75	0.4374	0.1814	0.2817	0.1864
	ClusterONE	1,016	338	0.5797	0.2678	0.3940	0.2831	436	0.5203	0.3983	0.4552	**0.2897**
	NCMine	3,263	1007	**0.5991**	0.1032	0.2487	0.2414	951	0.4603	0.1515	0.2641	0.2787
	SNFM	861	190	0.5124	0.1817	0.3051	0.1891	239	0.4711	0.2378	0.3347	0.1880
	PCNMTF	852	276	0.3597	0.3142	0.3362	0.2814	239	0.3479	0.3819	0.3645	0.2155
	svdcnmf	529	192	0.4123	0.1757	0.2691	0.2245	201	0.4246	0.2677	0.3371	0.2123
	NSSNMTF	724	277	0.5076	0.3076	0.3951	0.2891	349	0.5274	**0.4203**	**0.4708**	0.2834
	OSSNMTF	939	359	0.5026	0.3129	**0.3965**	**0.2961**	367	**0.5355**	0.4036	0.4649	0.2870
Mint	PCNMF	793	190	0.2739	0.2477	0.2605	0.2244	179	0.2983	0.3484	0.3224	0.1956
	CPSNMF	719	139	**0.6018**	0.1456	0.2960	0.1674	187	**0.5234**	0.2429	0.3566	0.1838
	PCSC	601	76	0.3344	0.1044	0.1869	0.1207	100	0.3318	0.1414	0.2166	0.1189
	ClusterONE	1,307	275	0.3081	**0.3088**	0.3084	**0.2311**	342	0.3311	**0.4304**	0.3775	0.2335
	NCMine	1,335	388	0.3050	0.1301	0.1992	0.2215	290	0.2990	0.1353	0.2011	0.1670
	SNFM	812	205	0.2801	0.2385	0.2585	0.2196	186	0.3059	0.3299	0.3176	0.2015
	PCNMTF	665	212	0.3734	0.2572	0.3099	0.2232	250	0.4556	0.3346	0.3905	0.2368
	svdcnmf	524	142	0.2749	0.2512	0.2628	0.2244	131	0.3059	0.3299	0.3176	0.1979
	NSSNMTF	647	198	0.4343	0.2723	**0.3439**	0.2307	250	0.4882	0.3771	**0.4291**	0.2410
	OSSNMTF	872	255	0.4214	0.2408	0.3185	0.2300	289	0.4935	0.3102	0.3912	**0.2420**
Mentha	PCNMF	876	217	0.3981	0.2667	0.3258	0.2144	222	0.3565	0.3220	0.3388	0.1819
	CPSNMF	995	175	**0.7043**	0.1313	0.3041	0.1669	275	**0.6826**	0.1842	0.3546	0.1693
	PCSC	960	98	0.1688	0.1637	0.1662	0.1462	92	0.2323	0.2194	0.2257	0.1259
	ClusterONE	1,813	403	0.4314	**0.3214**	0.3723	0.2347	592	0.3276	**0.4295**	0.3751	0.2127
	NCMine	11,961	2815	0.6316	0.0893	0.2374	0.2414	2012	0.5316	0.1073	0.2389	0.2293
	SNFM	912	229	0.4106	0.2579	0.3254	0.2144	235	0.3571	0.3310	0.3438	0.1890
	PCNMTF	872	261	0.4987	0.2696	0.3666	0.2213	335	0.4985	0.3411	0.4124	0.2185
	svdcnmf	763	196	0.4012	0.2701	0.3292	0.2216	202	0.3601	0.3314	0.3455	0.1871
	NSSNMTF	855	246	0.5037	0.2713	0.3697	0.2412	351	0.5454	0.3601	**0.4432**	0.2323
	OSSNMTF	978	296	0.5292	0.2797	**0.3847**	**0.2510**	397	0.5326	0.3621	0.4392	**0.2416**
STRING	PCNMF	914	274	0.4869	0.2887	0.3749	0.2590	266	0.4186	0.3572	0.3867	0.2091
	CPSNMF	842	178	0.5713	0.2285	0.3613	0.2103	256	0.5084	0.2881	0.3827	0.1911
	PCSC	932	93	0.1689	0.1553	0.1620	0.1436	103	0.2259	0.2075	0.2165	0.1288
	ClusterONE	1,800	300	0.5763	0.2068	0.3452	0.2139	480	0.4513	0.2858	0.3591	0.2002
	NCMine	7,838	2447	**0.8267**	0.1035	0.2925	0.2644	2046	**0.6189**	0.1403	0.2946	0.2313
	SNFM	926	281	0.4910	0.2889	0.3767	0.2603	272	0.4307	**0.3732**	0.4009	0.2103
	PCNMTF	813	259	0.5012	0.2802	0.3748	0.2691	264	0.4987	0.2881	0.3790	0.2219
	svdcnmf	580	205	0.4281	0.2792	0.3457	0.2349	214	0.4483	0.3785	0.4119	0.1663
	NSSNMTF	796	269	0.5119	0.2855	0.3823	**0.2789**	323	0.5278	0.3693	**0.4415**	0.2402
	OSSNMTF	996	364	0.5340	**0.2920**	**0.3949**	0.2750	408	0.5630	0.3167	0.4223	**0.2434**
WSTRING	PCNMF	902	271	0.4867	0.2832	0.3713	0.2583	262	0.4307	0.3460	0.3860	0.2094
	CPSNMF	815	181	0.5544	0.2301	0.3571	0.2076	253	0.5150	0.2863	0.3840	0.1909
	PCSC	126	16	0.5784	0.0905	0.2288	0.1676	31	0.5279	0.1014	0.2314	0.1726
	ClusterONE	1,474	256	**0.5998**	0.1926	0.3399	0.1948	385	0.5079	0.2523	0.3580	0.1818
	NCMine	-	-	-	-	-	-	-	-	-	-	-
	SNFM	897	274	0.4951	0.2817	0.3734	0.2596	287	0.4267	0.3532	0.3882	0.2127
	PCNMTF	900	319	0.5135	0.2781	0.3779	0.2541	352	**0.5623**	0.3038	0.4133	0.2207
	svdcnmf	660	209	0.5115	0.2591	0.3640	0.2533	199	0.4392	0.3008	0.3635	0.1751
	NSSNMTF	786	271	0.5054	0.2826	0.3780	0.2723	341	0.5278	**0.3728**	**0.4436**	**0.2496**
	OSSNMTF	996	367	0.5299	**0.2923**	**0.3936**	**0.2765**	396	0.5590	0.3227	0.4247	0.2380

*Bold means the best value*.

**Figure 7:**
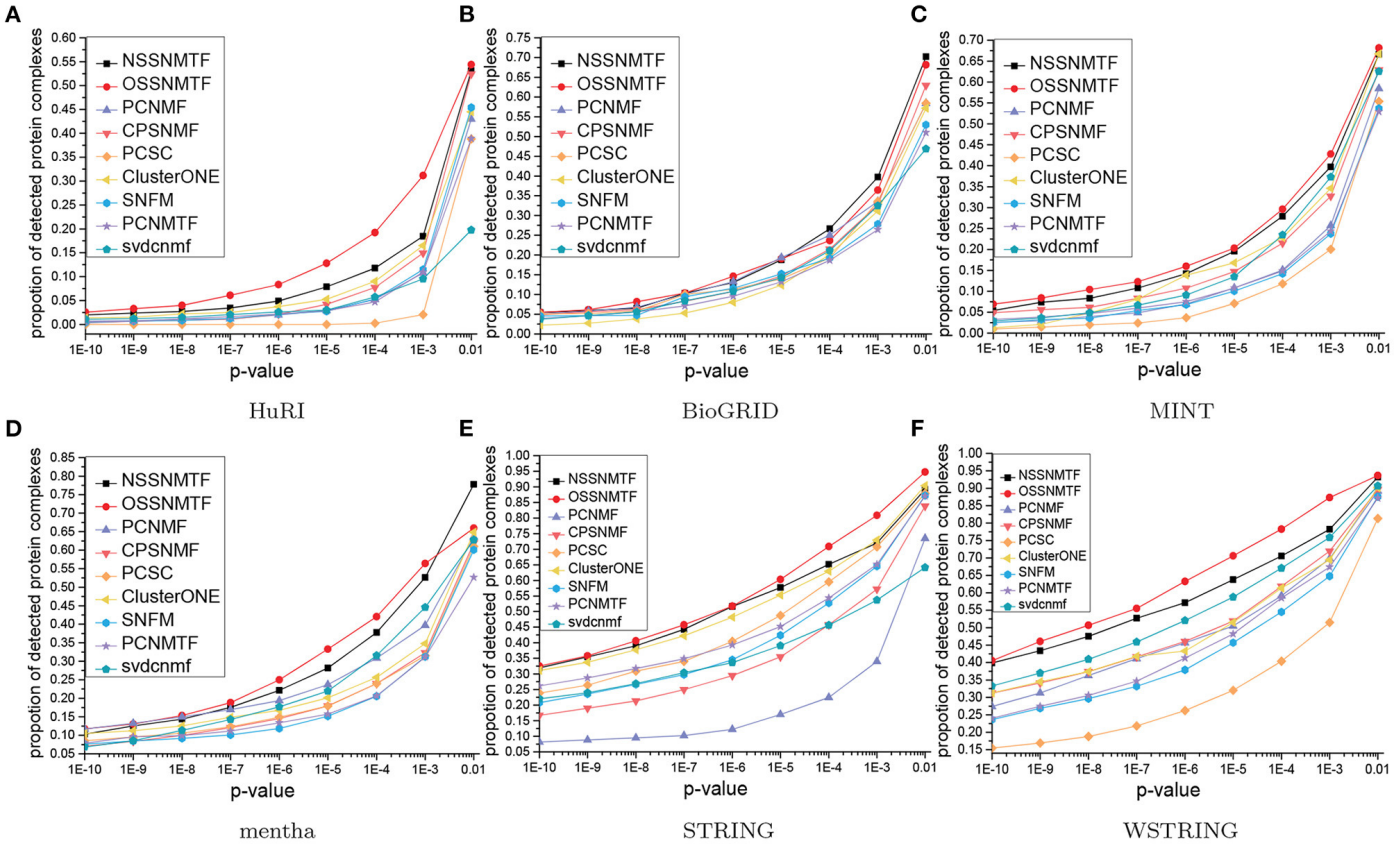
*P*-value proportion distributions of significant detected protein complexes with respect to BP. The *x*-axis represents *p*-values, *y*-axis denotes the proportion of protein complexes. **(A)** HuRI; **(B)** BioGRID; **(C)** MINT; **(D)** mentha; **(E)** STRING; **(F)** WSTRING.

To further probe the functional homogeneities of detected protein complexes, gene ontology (GO) (Consortium, 2019) data were used to assess the biological roles of these protein complexes. We conducted enrichment analysis on all detected protein complexes in terms of the GO sub-ontology category denoted as “biological process" (BP), using *p*-values with false discovery rates. These results imply the enrichment of a specific GO term in a single protein complex. Thus, the detected protein complexes were presumed to have greater biological importance when they exhibited smaller *p*-values. The proportions of protein complexes with *p*-values below a given threshold can represent the performance of protein complex detection algorithms. In this paper, we varied the threshold from 10^−10^ to 0.01, then measured the proportions of functionally enriched protein complexes in each threshold interval for each algorithm. A higher proportion indicated better performance of the corresponding algorithm in the detection of protein complexes from PPI networks. Figure 7 shows the *p*-value proportion distributions for all compared algorithms with respect to BP. The proposed method NSSNMTF outperformed other non-overlapping protein complex detection methods (i.e., 66.62% of protein complexes had *p* ≤ 0.01 in terms of BP on MINT), while OSSNMTF outperformed other overlapping protein complex detection methods (i.e., 68.15% of protein complexes had *p* ≤ 0.01 in terms of BP on MINT). Overall, the protein complexes detected by the proposed models NSSNMTF and OSSNMTF had greater probabilities of predicting real protein complexes.

To further explore the biological significance of detected protein complexes, the top six enriched protein complexes provided by NSSNMTF based on BioGRID PPI database are presented is Table 4. We listed the size of protein complex, GO ID, *p*-value and GO term, respectively. We can find that the *p*-values of the six protein complexes are very low which means they have higher probabilities to be regarded as real complexes. As the *p*-value is closed to the size of protein complex, and the *p*-value of the small protein complex is usually high. Therefore, the second column of Table 4 shows the sizes of enriched protein complexes. From this column, we can find that our detected protein complexes with big size can match well with the gene ontology term. This means the biological significance of protein complexes detected by our model is high, and that indicates that the protein complexes predicted by our proposed model have greater probability to be true protein complexes.

**Table 4:** The top 6 enriched protein complexes based on BioGRID PPI network in terms of BP.

**ID**	**Size**	**GO ID**	***p*-value**	**GO term**
1	79	GO:0006412	3.45E-93	Translation
2	54	GO:0006120	1.57E-68	Mitochondrial electron transport, NADH to ubiquinone
3	132	GO:0000398	3.03E-55	mRNA splicing, via spliceosome
4	14	GO:0006385	5.71E-32	Transcription elongation from RNA polymerase III promoter
5	14	GO:0006415	5.73E-29	Translational termination
6	20	GO:0033572	1.68E-27	Transferrin transport

**Figure 8:**
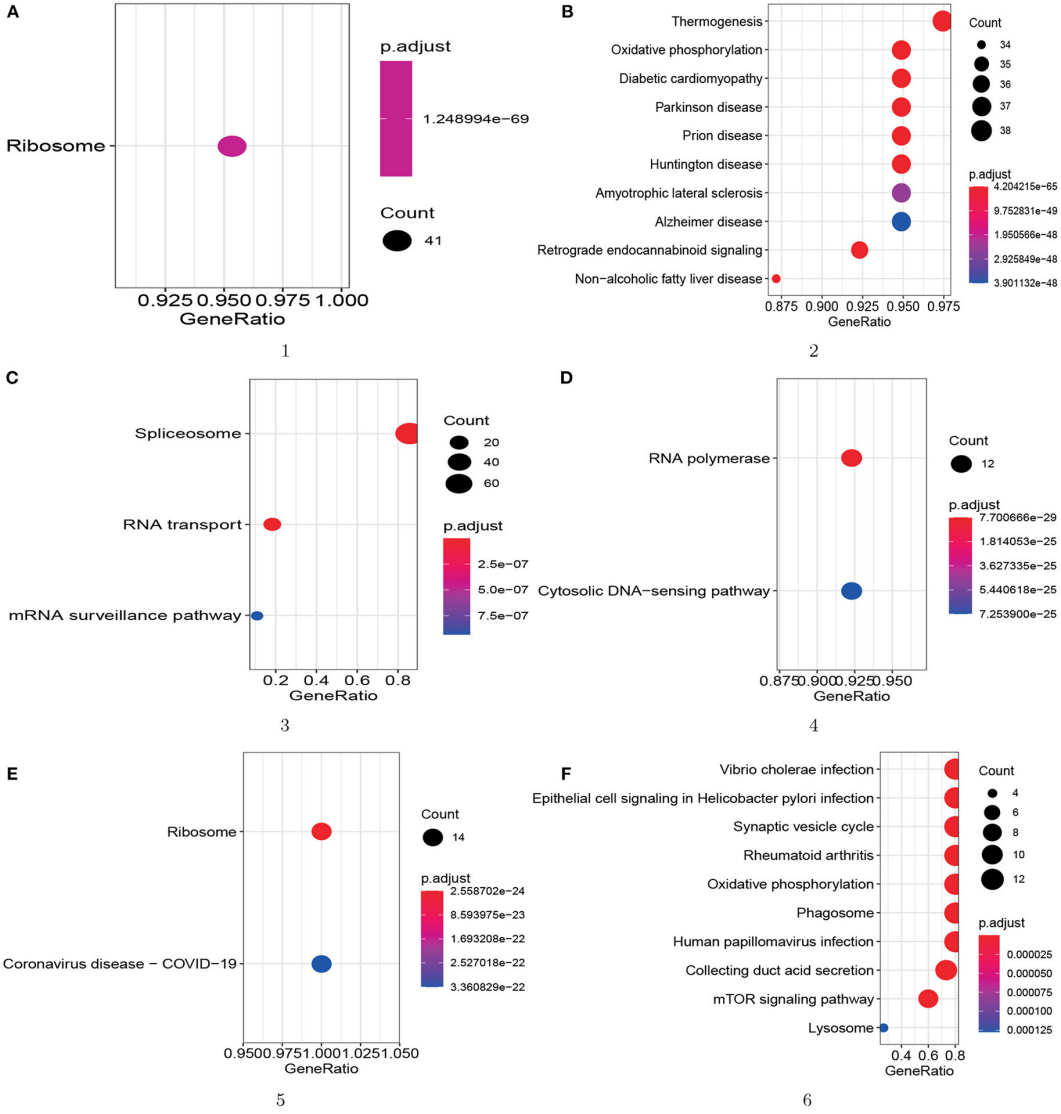
KEGG pathway analysis for the top 6 protein complexes enriched on BP. The GeneRatio is the number of proteins annotated by a specific pathway divided by the number of proteins annotated by any pathway. The size of the circle represents the number of proteins annotated by one pathway. **(A)** module 1; **(B)** module 2; **(C)** module 3; **(D)** module 4; **(E)** module 5; **(F)** module 6.

Furthermore, to better demonstrate the biological meaning of our detected protein complex, we do KEGG pathway analysis for all six of the protein complexes. The results are showed in Figure 8, and we can find that all six of the predicted protein complexes are enriched on one or more pathways with very small *p*-values based on KEGG database. The result shows that these six protein complexes enriched on BP also have high biological significance on pathways. More importantly, we found that all 14 proteins (RPL10, RPL21, RPL23, RPS3A, RPL9, RPS4X, RPS26, RPS25, RPL7A, RPL37A, RPS3, RPL14, RPL36, and RPL19) in the fifth protein complex (ID=5) are annotated by the COVID-19 pathway from Figure 8E. Then this predicted protein complex can provide molecular support for the treatment mechanism and drug development of COVID-19. Therefore, the KEGG pathway enrichment results illustrate that our proposed model is reliable for protein complex detection.

### 3.4. Case Study

**Figure 9:**
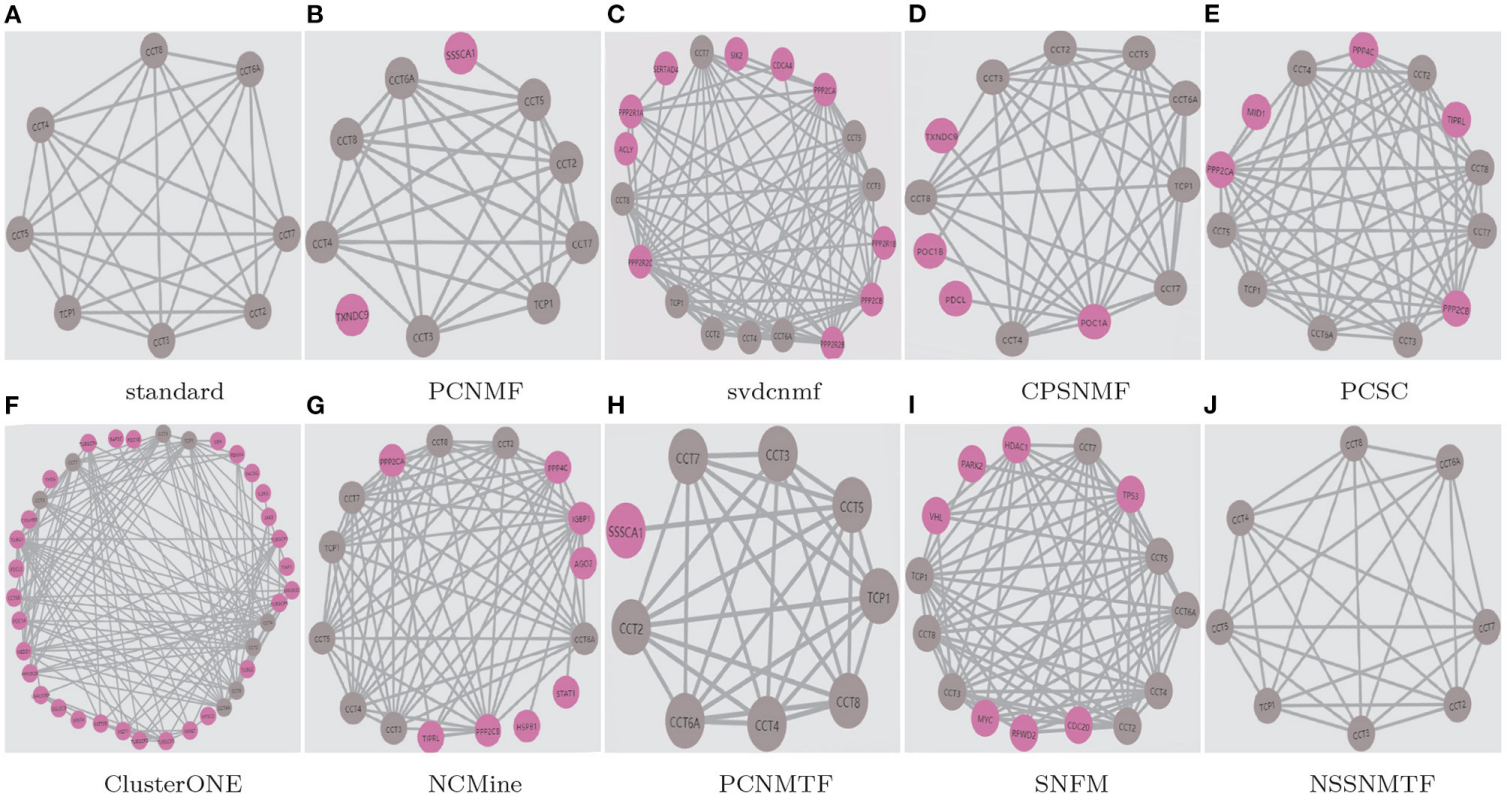
The chaperonin containing TCP1 complex detected by different algorithms based on BioGRID PPI network. Standard means the true protein complex. True positive proteins are shown in gray. False positive proteins are shown in pink. False negative proteins are shown in green. **(A)** standard; **(B)** PCNMF; **(C)** svdcnmf; **(D)** CPSNMF; **(E)** PCSC; **(F)** ClusterONE; **(G)** NCMine; **(H)** PCNMTF; **(I)** SNFM; **(J)** NSSNMTF.

**Figure 10:**
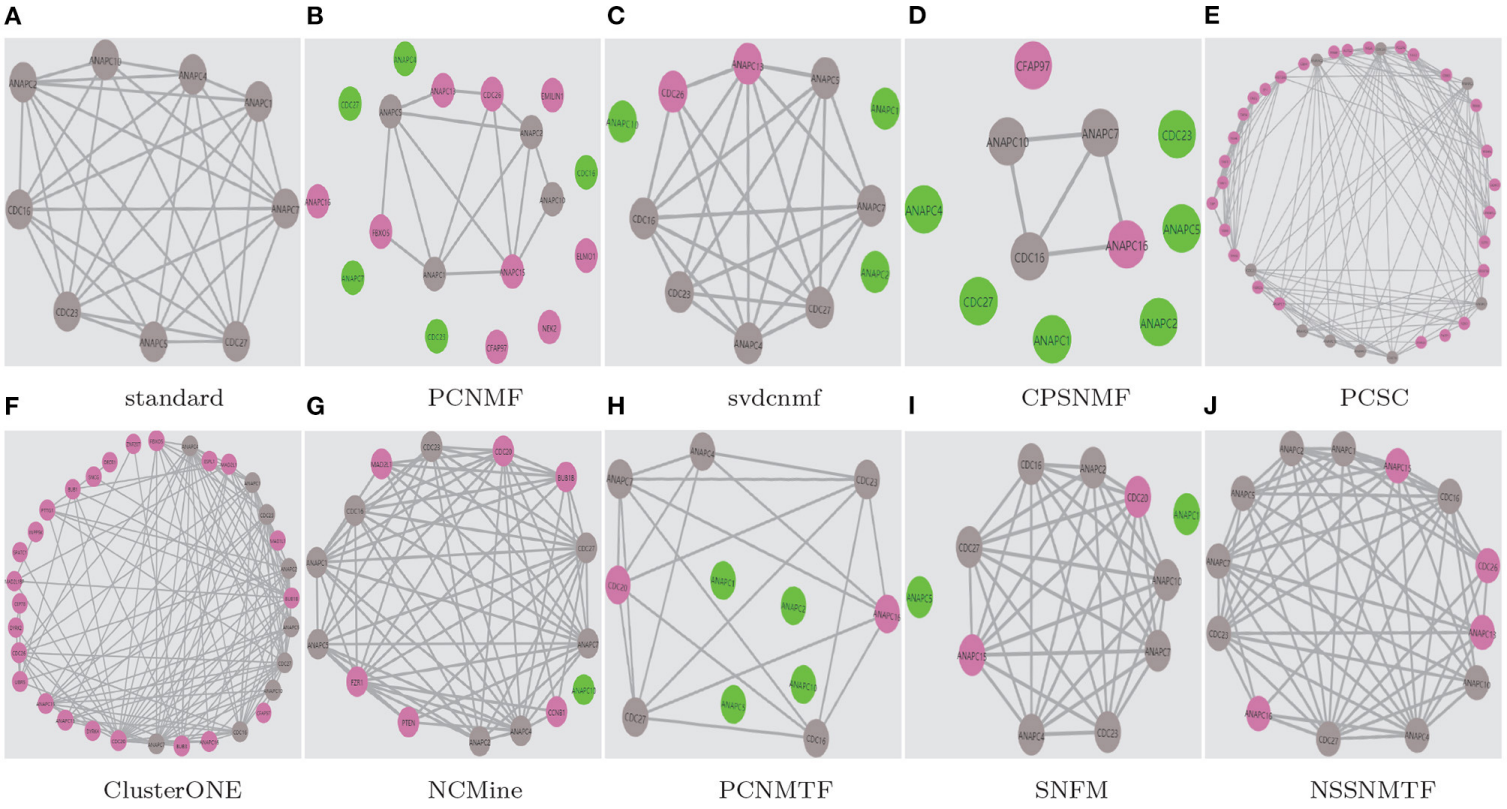
The Anaphase-promoting complex detected by different algorithms based on BioGRID PPI network. Standard means the true protein complex. True positive proteins are shown in gray. False positive proteins are shown in pink. False negative proteins are shown in green. **(A)** standard; **(B)** PCNMF; **(C)** svdcnmf; **(D)** CPSNMF; **(E)** PCSC; **(F)** ClusterONE; **(G)** NCMine; **(H)** PCNMTF; **(I)** SNFM; **(J)** NSSNMTF.

To demonstrate the superiority of our proposed model, two well-studied protein complexes (chaperonin containing TCP1 complex Li et al., 2021 and Anaphase-promoting complex Li et al., 2020) are chosen as examples in this paper. Figures 9, 10 show the detected results of different algorithms on the two protein complexes from BioGRID PPI network, respectively.

The proposed NSSNMTF and OSSNMTF give the same predicted results on identifying these two protein complexes. Obviously, on identifying chaperonin containing TCP1 complex, all the compared algorithms cover all the 8 standard proteins and predict one or more false positive proteins, but the protein complexes detected by our proposed model NSSNMTF match the standard protein complex exactly. PCNMTF, PCNMF, CPSNMF, PCSC, SNFM, NCMine, svdcnmf, and ClusterONE predict 1, 2, 4, 5, 7, 8, 10, and 30 false positive proteins, respectively. Specifically, for the Anaphase-promoting complex, NSSNMTF, ClusterONE, and PCSC cover all the 9 standard proteins, and predict 4, 24, and 28 false positive proteins, respectively. NCMine covers 8 out of the 9 standard proteins and predict 6 false positive proteins and 1 false negative protein. PCNMF and CPSNMF cover 4 and 3 out of 9 standard proteins that means they fail to detect the Anaphase-promoting complex. SNFM covers 7 out of the 9 standard proteins and predicts 2 false positive proteins and 2 false negative proteins. svdcnmf covers 6 out of the 9 standard proteins and predicts 2 false positive proteins and 3 false negative proteins. PCNMTF covers 5 out of the 9 standard proteins and predicts 2 false positive proteins and 4 false negative proteins. Although the number of false positive standard proteins predicted by these 3 algorithms is 2, they all missed some standard proteins. In conclusion, our proposed model detects fewer false positive proteins to protein complexes without detecting false negative standard proteins, and indicates the best performance.

## 4. Discussion

In this paper, we proposed the SSNMTF semi-supervised protein complex detection model, which can simultaneously learn protein membership and module relationship matrices by using pairwise constraints as prior information from weighted and unweighted PPI networks. We also proposed two initial approaches for the two variables in SSNMTF and two parameter-free protein complex detection methods: a non-overlapping module detection algorithm (NSSNMTF) and an overlapping protein complex detection algorithm (OSSNMTF). We have conducted experiments on both synthetic networks and human PPI networks to evaluate the performances of our proposed algorithms, and the experimental results showed that SSNMTF is superior to all compared algorithms. These findings indicate that the use of high-quality prior information can help to efficiently discover important biological protein complexes with clear module structures. In our future work, we will simultaneously consider protein complexes and GO information when mining protein complexes from human PPI networks.

## Data Availability Statement

The datasets presented in this study can be found in online repositories. The names of the repository/repositories and accession number(s) can be found below: https://dip.doe-mbi.ucla.edu/dip/Main.cgi, https://string-db.org/.

## Author Contributions

GL, XZ, and JY conceived the study, carried out its design and all experiments, and drafted the manuscript. BL, AL, and XW helped draft the manuscript and participated in its design and coordination. All the authors read and approved the final manuscript.

## Conflict of Interest

The authors declare that the research was conducted in the absence of any commercial or financial relationships that could be construed as a potential conflict of interest.

## Publisher's Note

All claims expressed in this article are solely those of the authors and do not necessarily represent those of their affiliated organizations, or those of the publisher, the editors and the reviewers. Any product that may be evaluated in this article, or claim that may be made by its manufacturer, is not guaranteed or endorsed by the publisher.
